# Q Fever, Scrub Typhus, and Rickettsial Diseases in Children, Kenya, 2011–2012

**DOI:** 10.3201/eid2205.150953

**Published:** 2016-05

**Authors:** Alice N. Maina, Christina M. Farris, Antony Odhiambo, Ju Jiang, Jeremiah Laktabai, Janice Armstrong, Thomas Holland, Allen L. Richards, Wendy P. O’Meara

**Affiliations:** Naval Medical Research Center, Silver Spring, Maryland, USA (A.N. Maina, C.M. Farris, A. Odhiambo, J. Jiang, A.L. Richards);; Moi University College of Health Sciences, Eldoret, Kenya (J. Laktabai, J. Armstrong, W.P. O’Meara);; Duke Global Health Institute and Duke University School of Medicine, Durham, North Carolina, USA (T. Holland, W.P. O’Meara)

**Keywords:** Q fever, Coxiella burnetii, scrub typhus, rickettsial diseases, rickettsiae, spotted fever group rickettsia, typhus group rickettsia, Kenya, pediatric, undifferentiated fever, febrile illness, bacteria

## Abstract

To increase knowledge of undifferentiated fevers in Kenya, we tested paired serum samples from febrile children in western Kenya for antibodies against pathogens increasingly recognized to cause febrile illness in Africa. Of patients assessed, 8.9%, 22.4%, 1.1%, and 3.6% had enhanced seroreactivity to *Coxiella burnetii*, spotted fever group rickettsiae, typhus group rickettsiae, and scrub typhus group orientiae, respectively.

Rickettsial diseases, scrub typhus, and Q fever are increasingly recognized as key causes of undifferentiated fevers in humans in Africa (*1*–*6*). According to a recent study in western Kenya, respiratory viral infections were responsible for ≈41% of all fevers in children, but 37.1% were of unknown etiology. In the same study, malaria accounted for 5.2% of fevers (*7*). Several attempts have been made to elucidate causes of febrile illnesses in Kenya, but none have focused on causes among children, particularly in settings in which animal husbandry is integrated into communities. To further knowledge of the causes of febrile illnesses in western Kenya, we tested paired acute- and convalescent-phase serum samples from febrile children.

## The Study

Participants were febrile, 1- to 12-year-old children brought for care at Webuye District Hospital (WDH; Bungoma, Kenya). Details of patient recruitment were previously reported (*7*). In brief, children with fever >37.5°C who lived within the administrative boundaries of Webuye Division were enrolled after informed consent was obtained. Acute-phase serum samples were stored at the time of enrollment. Patients returned ≈4 weeks after enrollment for follow-up physical examination and collection of convalescent-phase serum samples. The study protocol was approved by the Moi University Research and Ethics Committee (Eldoret, Kenya), WDH, and the Duke University Institutional Review Board (Durham, North Carolina, USA).

We used ELISAs as described (*5*,*8*–*10*) to evaluate the serum samples for IgG against spotted fever group rickettsiae (SFGR), typhus group rickettsiae (TGR), and scrub typhus group orientiae (STGO). In brief, all convalescent-phase and any unpaired acute-phase serum samples were screened (1:100 dilution), and screen-positive samples were titered in parallel with the corresponding acute-phase samples by serial dilution to assess enhanced seroreactivity to group-specific antigens. Enhanced seroreactivity was defined as a seroconversion from nonreactive in the acute phase to reactive (titer >400) in the convalescent phase or as a 4-fold rise in antibody titer between acute- and convalescent-phase serum samples. Results for samples that showed enhanced seroreactivity for STGO by ELISA were further confirmed by Western blot, using the recombinant proteins Kpr56 (*9*) and Kpr47b (*5*). The Kpr47b Western blot assay was performed as described (*5*) with the following modifications: Kpr47 antigen was loaded in a Mini-PROTEAN TGX Precast Gel and separated in a Mini Tetra cell electrophoresis module (both from Bio-Rad, Hercules, CA, USA). After being blocked for 1 hour, the membranes were incubated in serum samples diluted 1:100 in 10% blocking buffer and polyclonal *Escherichia coli* protein (ratio 1:1). Pierce ECL Plus Western Blotting Substrate (ThermoFisher Scientific, Waltham, MA, USA) was used to develop blots; signal was detected on a ChemiDoc XRS+ System (Bio-Rad).

To screen convalescent-phase serum samples, we used a *Coxiella burnetii* ImmunoDot assay (GenBio, San Diego, CA, USA) according to the manufacturer’s instructions. Samples were diluted 1:200 in the initial stage of this immunoassay.

Data were imported into Stata 11.2 (StataCorp LP, College Station, TX, USA) for analysis. We used χ^2^ tests to compare categorical variables across groups, and we applied *t*-tests for continuous variables; p<0.05 was considered significant.

A total of 370 febrile children were enrolled in the study during November 2011–December 2012. The average age was 4.4 (SD 2.8) years; 48.4% of enrollees were boys. The main symptom at first examination was fever (mean temperature 38.2°C [SD 0.6°C]). Most children had been ill for 2 days before arrival at WDH, and 20% had been pretreated with an antimalarial drug, an antimicrobial drug, or both. Average time between enrollment and follow-up was 45 (SD 12) days. Overall, 364 convalescent- and unpaired acute-phase serum samples were screened; 47 (12.9%), 104 (28.6%), 6 (1.6%), and 21 (5.8%) were seropositive for *C. burnetii*, SFGR, TGR, and STGO, respectively. Of the 364 serum samples, 281 (77.2%) represented paired acute- and convalescent-phase samples, of which 25 (8.9%), 63 (22.4%), 3 (1.1%), and 10 (3.6%) had results indicative of acute *C. burnetii,* SFGR, TGR, and STGO infection, respectively (Table 1). Endpoints were 400 to >6,400 for SFGR, 400 to 1,600 for STGO, and 400 for TGR. Dual infections were noted in 27 (9.6%) of the 281 paired acute- and convalescent-phase samples: STGO and SFGR were found in 7 (2.5%), *C. burnetii* and SFGR in 14 (5.0%), *C. burnetii* and TGR in 2 (0.7%), and *C. burnetii* and STGO in 4 (1.4%) samples.

**Table 1:** Prevalence of IgG against *Coxiella burnetii*, SFGR, TGR, and STGO in serum samples from febrile children attending Webuye District Hospital, Bungoma, Kenya, November 2011–December 2012*

Antigen type	Total no. samples screened	No. (%) screen positive	Total no. paired samples	No. (%) IgG-positive†	No. (%) acute infections‡
*C. burnetii*	364	47 (12.9)	281	10 (3.6)	25 (8.9)
SFGR	364	104 (28.6)	281	23 (8.2)	63 (22.4)
TGR	364	6 (1.6)	281	1 (0.4)	3 (1.1)
STGO	364	21 (5.8)	281	9 (3.2)	10 (3.6)

*The children were 1–12 years of age. SFGR, spotted fever group rickettsiae; STGO, scrub typhus group orientiae; TGR, typhus group rickettsiae. †No change in antibody titer between acute- and convalescent-phase serum samples. ‡Seroconversion or 4-fold rise in antibody titer.

To ensure no cross reactivity of *C. burnetii*– and STGO-positive serum samples, we used Western blot to test the *C. burnetii*–positive serum against STGO proteins. No cross-reactivity was observed with the *C. burnetii*–specific IgG–positive serum and the *Orientia*-specific recombinant proteins (Figure).

**Figure:**
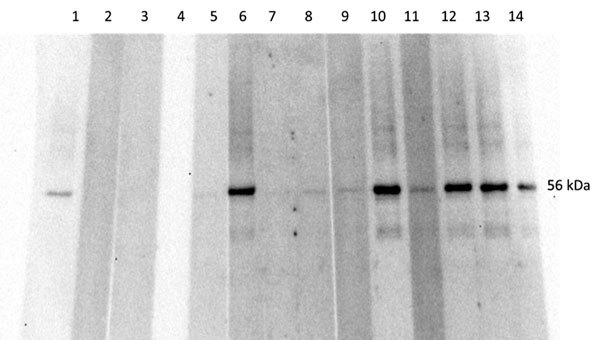
Western blot analysis, using *Orientia* 56Kpr recombinant protein, of serum samples from febrile children in western Kenya, November 2011–December 2012. Lane 1, positive control; lane 2, negative control; lanes 3–4, *Coxiella burnetii*–positive patients; lane 5, *Orientia* spp.–negative patient; lanes 6–14, *Orientia* spp.–positive patients.

Statistical analyses showed SFGR-infected children were more likely to be girls than boys (p = 0.007), indicating sex as a possible risk factor (Table 2). The primary clinical signs of all infections were cough, diarrhea, vomiting, and inability to drink or breastfeed. *C. burnetii*–infected children were more likely than *C. burnetii*–uninfected children to report cough (p = 0.018). Of the children with rickettsial infections, 81%–90% received antimalarial drugs and ≈60% received antimicrobial drugs; similar proportions were seen for Q fever patients. Virtually all Q fever and rickettsia-infected patients received a diagnosis of malaria by the treating clinician. *C. burnetii* and SFGR cases were detected throughout the year, with peaks noted in February–June for SFGR and April–July for *C. burnetii*.

**Table 2:** Analysis of variables across *Coxiella burnetii*, SFGR, and STGO infections in febrile children attending Webuye District Hospital, Bungoma, Kenya, November 2011–December 2012*

Variable	*C. burnetii*				SFGR				STGO		
	Positive, n = 25†	Negative, n = 256†	p value		Positive, n = 63†	Negative, n = 218†	p value		Positive, n = 10†	Negative, n = 271†	p value
Mean age, y (SD)	3.8 (0.48)	4.5 (0.18)	0.283		3.8 (0.35)	4.6 (0.19)	0.065		4.8 (0.87)	4.4 (0.17)	0.669
Sex											
M	39	47	0.452		31.8	51.1	0.007‡		20	47.6	0.086
F	61	52			68.2	48.9			80	52.4	
Symptom											
Rash	0	1.6	0.548		1.6	1.4	0.901		0	1.5	0.7
Vomiting	21.7	16.7	0.536		25.4	14.7	0.046§		30	16.6	0.269
Cough	73.9	48	0.018#		54	49.1	0.495		60	49.8	0.527
Diarrhea	17.4	10.5	0.31		15.9	9.6	0.164		30	10.3	0.051
Difficulty breathing	0	1.6	0.548		1.6	1.4	0.901		0	1.5	0.7
Inability to drink or breastfeed	13	7.4	0.331		9.5	7.3	0.57		10	7.8	0.795
Admitted to hospital	30.4	23.6	0.466		20.6	25.2	0.453		10	24.7	0.286
Treatment administered at hospital											
Antimicrobial drug	73.9	54.7	0.074		61.9	59.7	0.746		50	57.2	0.505
Antimalarial drug	82.6	81	0.851		81	81.2	0.966		90	80.8	0.466
Recovered after treatment	100	92.6	0.158		98.4	92.3	0.076		100	92.7	0.372
Malaria diagnosed by clinician	100	89.8	0.094		95.2	89.1	0.138		100	90.2	0.294

*SFGR, spotted fever group rickettsiae, STGO, scrub typhus group orientiae.  †Values are percentages unless otherwise specified. ‡Being a girl was indicated as a possible risk factor for SFGR infection (p<0.05). §Children with SFGR were more likely to report vomiting (p<0.05). #Children with *C. burnetii* were more likely to report cough (p<0.05).

## Conclusions

In this study, the seroprevalence of IgG against SFGR was 28.7%, a finding similar to that in a previous study in Tanzania (*11*). We observed higher rates (22.4%) of acute SFGR infections than were observed among pediatric patients in Tanzania (>8%), where similar serologic tools were used to diagnose acute rickettsial infections (*11*,*12*). Acute SFGR infections occurred throughout the year, but increases were noted during February–June, months which coincide with the long rainy season in Kenya. Seasonal peaks of SFGR infections coinciding with wetter months have been observed in sub-Saharan Africa (*3*). A higher frequency of acute SFGR was noted in girls than boys and may be related to occupational exposure, as noted among women in Peru (*13*). TGR rates in our study are comparable to those reported in Tanzania (*11*). 

The seroprevalence of STGO was 5.8%, similar to that reported from a previous study in Kenya (*5*). Detection of enhanced seroreactivity to scrub typhus suggests acute infections with *Orientia* spp. Scrub typhus was formerly thought to be geographically restricted to Asia, but the distribution has been redefined by the recent detection of *Orientia* DNA in mice in Africa; description of scrub typhus–like illnesses in Chile and United Arab Emirates; and discovery of a new species, *O. chuto,* isolated from a patient who had visited Dubai (*14*). 

In addition, the prevalence of antibodies to *C. burnetii* observed in our study is higher (12.9%) than that reported for other African countries (<8%) but within the range reported among children in Egypt (10%–32%) (*15*). Our findings showed a much higher rate (8.9%) of acute Q fever than reported among infants and children in Tanzania (2.6%) (*11*). A distinct seasonality was noted with acute Q fever infections, and this may be related to the parturient season in domestic and wild animals (*11*). The dual infections with SFGR and STGO and STGO and *C. burnetii* may be the result of similar risk factors. Additional research is needed to identify the reservoirs and vectors of *Orientia* spp. in Africa and to identify key risk factors for infection.
